# Electrochemical aptasensor based on the engineered core-shell MOF nanostructures for the detection of tumor antigens

**DOI:** 10.1186/s12951-023-01884-5

**Published:** 2023-04-26

**Authors:** Suliman Khan, William C. Cho, Afrooz Sepahvand, Sara Haji Hosseinali, Arif Hussain, Mohammad Mahdi Nejadi Babadaei, Majid Sharifi, Mojtaba Falahati, Laila Abdulmohsen Jaragh-Alhadad, Timo L. M. ten Hagen, Xin Li

**Affiliations:** 1 Medical Research Center, The Second Affiliated Hospital of Zhengzhou University, Zhengzhou, China; 2 Department of Medical Lab Technology, The University of Haripur, Haripur, Pakistan; 3 Department of Clinical Oncology, Queen Elizabeth Hospital, Kowloon, Hong Kong China; 4 Department of Cellular and Molecular Biology, Faculty of Advanced Science and Technology, Tehran Medical Sciences, Islamic Azad University, Tehran, Iran; 5 Department of Genetics, Faculty of Advanced Science and Technology, Tehran Medical Sciences, Islamic Azad University, Tehran, Iran; 6 School of Life Sciences, Manipal Academy of Higher Education, Dubai, United Arab Emirates; 7 Department of Molecular Genetics, Faculty of Biological Science, North Tehran Branch, Islamic Azad University, Tehran, Iran; 8 Student Research Committee, School of Medicine, Shahroud University of Medical Sciences, Shahroud, Iran; 9 Depatment of Tissue Engineering, School of Medicine, Shahroud University of Medical Sciences, Shahroud, Iran; 10 Precision Medicine in Oncology (PrMiO), Department of Pathology, Erasmus MC Cancer Institute, Erasmus MC, Rotterdam, The Netherlands; 11 Nanomedicine Innovation Center Erasmus (NICE), Erasmus MC, Rotterdam, The Netherlands; 12 Department of Chemistry, College of Science, Kuwait University, 13060 Safat, Kuwait; 13 Department of Neurology, The Second Affiliated Hospital of Zhengzhou University, Zhengzhou, China

**Keywords:** Core-shell, Metal organic framework, Aptasensors, Cancer biomarkers

## Abstract

It is essential to develop ultrasensitive biosensors for cancer detection and treatment monitoring. In the development of sensing platforms, metal-organic frameworks (MOFs) have received considerable attention as potential porous crystalline nanostructures. Core-shell MOF nanoparticles (NPs) have shown different diversities, complexities, and biological functionalities, as well as significant electrochemical (EC) properties and potential bio-affinity to aptamers. As a result, the developed core-shell MOF-based aptasensors serve as highly sensitive platforms for sensing cancer biomarkers with an extremely low limit of detection (LOD). This paper aimed to provide an overview of different strategies for improving selectivity, sensitivity, and signal strength of MOF nanostructures. Then, aptamers and aptamers-modified core-shell MOFs were reviewed to address their functionalization and application in biosensing platforms. Additionally, the application of core-shell MOF-assisted EC aptasensors for detection of several tumor antigens such as prostate-specific antigen (PSA), carbohydrate antigen 15-3 (CA15-3), carcinoembryonic antigen (CEA), human epidermal growth factor receptor-2 (HER2), cancer antigen 125 (CA-125), cytokeratin 19 fragment (CYFRA21-1), and other tumor markers were discussed. In conclusion, the present article reviews the advancement of potential biosensing platforms toward the detection of specific cancer biomarkers through the development of core-shell MOFs-based EC aptasensors.

## Introduction

Biomarker detection plays a major role in the timely diagnosis of a wide range of disorders, including neurodegenerative, autoimmune and cancer diseases. The discovery of cancer biomarkers may lead to the early detection of cancer, which in turn will significantly impact reducing cancer-related mortality. Also, monitoring the treatment process, diagnosis, and application of an appropriate strategy for cancer therapy, assessment of disease status, drug production, and prescription of appropriate drugs can be done with the help of biomarkers.

As a result, biomedical researchers are increasingly focused on developing a feasible and accurate approach that will result in the sensitive detection of biomarkers. Currently, multiple analytical analyses have been applied for detecting cancer-related biomarkers including spectrophotometry [1, 2], electrophoresis [3, 4], liquid chromatography [5, 6], and sensor [7–9]. For example, surface-enhanced Raman spectroscopy nanoprobes have been widely used for the detection of cancer cells [10], exosomes [11, 12], and protein biomarkers [13].

Furthermore, two-dimensional differential gel electrophoresis has been demonstrated as a promising platform for the detection of various types of cancer biomarkers with high reproducibility and sensitivity [14]. Furthermore, liquid chromatography coupled to mass spectrometry has been broadly utilized for the potential detection of cancer biomarker peptides [15], plasma lipid profile [16], splicing biomarkers [17], and modified nucleosides [18]. Also, different types of biosensors including EC-, optical-, and mass-based techniques have been used to detect cancer biomarkers [7, 19, 20]. EC-based biosensing assays stand out among different types of analytical methods and biosensors due to their fast reactivity, high sensitivity, easy operation, and cost-effectiveness. In the recent years, different types of nanomaterials including iron oxide NP bioconjugates [21], gold (Au) NP decorated multiwall carbon nanotubes- [22], sandwiched silver (Ag) NPs in N-doped graphene- [23], AuNPs/graphene quantum dots (QDs)/graphene oxide (GO) film- [24], and hierarchical flower-like molybdenum disulphide (MoS_2_) NP- [25] modified EC electrodes have been used to detect cancer biomarkers. However, some major drawbacks including narrow linear range and limited sensitivity still hinder their potential application in the biomedical field. Hence, it is inevitable to develop new electrocatalysts with high sensitivity and selectivity for the detection of cancer biomarkers.

Nonenzymatic-based biosensing approaches show several advantages in comparison with enzymatic-based biosensing strategies including a lower LOD, faster reactive times, improved long/short-term stability, and cost-effectiveness [26–28]. One of the most important strategies for promoting EC detection activity relies on the application of potential materials presenting high conductivity along with a large reactive surface area.

Following the introduction of metal-organic framework (MOF) with 3D periodic infinite network architectures fabricated through the coordination of metals and organic materials as ligands [29, 30], a large number of studies have been published on the synthesis and utilization of colloidal-sized MOF nanostructures [31–33]. In comparison to zeolite- and carbon-based materials, MOF NPs as porous materials exhibit several novel superiorities such as tunable pore dimensions, functionalized pore surfaces, ultralow density, and ultrahigh active surface areas [34], which endow MOFs with exclusive benefits in different applications, including biosensing [35, 36] and catalyst [37, 38]. Recently, there has been a lot of interest in using MOF NPs to develop potential biosensors for the detection of different biological or chemical reactions [39, 40].

Several MOF-based architectures, including nanowires, nanotubes, octahedral, and core-shell structures have been reported for application in different fields [41–43]. Among these structures, core-shell architectures have demonstrated significant advantages due to their highly appealing topologies and potential chemical activities [34]. In comparison to other MOF structures, the presence of a shell can result in the formation of a proportionally stable and unaffected microenvironment for catalytic reactions, as well as the combination of multiple properties via the synergistic feature between the core and shell units [34]. The primary core-shell MOF architectures are identified by a metal core covered with an MOF shell [44]. Nevertheless, there are several common core-shell structures, including metal and non-metal NPs@MOFs, MOF@metal oxide NPs, and MOFs@MOFs [34, 45, 46].

As a result, because several materials exhibit synergistic performance, the combination of different bio-functional compounds has been a hot topic in the biomedical field. Because of the unique architecture of core-shell MOFs, the developed electrodes exhibit promising long-term stability with boosted mechanical durability [47] and ultra-sensitive EC detection [48]. In fact, core-shell MOFs are used in a variety of biosensors with different detection methods, and have the advantages of exhibiting a rapid reactivity and reusability, as well as improved sensitivity, increased selectivity, and a feasible assay strategy [49, 50].

To date, electrochemistry-based immune assays have been reported for detecting tumor markers with a potential sensitivity and accuracy, a significantly low LOD, and a pronounced signal augmentation [51]. Although this immunoassay has high sensitivity and efficiency, the need for complicated washing steps and heterogeneous responses in this assay cause diverse antibody (Ab)-antigen interactions and diminished Ab performance [52, 53], which affect detection sensitivity and experimental reusability [53]. Aptamers, on the other hand, have different significant advantages over antibodies, including their small size, low cost, increased chemical stability, and feasible design [54, 55]. These characteristics have received widespread attention and have shown promise in addressing the aforementioned immunoassay concerns [56]. Aptamers have also demonstrated some benefits for developing potential biosensors with improved selectivity and sensitivity [57–59].

Therefore, developing potential core-shell MOF with unique structures and recruiting them as a solid support for the immobilization of aptamers can be used to detect different cancer markers. Indeed, when aptamer strands adsorbed onto the MOF platform interact with cancer biomarkers, the resulting conformational changes in the aptamer can be detected using various sensors, particularly EC-based platforms. Additionally, various MOF architectures result in different surface and chemical capabilities, changing the sensing potency of aptamers by regulating the interaction of redox ions with the electrode surface.

## Application of core-shell MOFs in the development of biosensing platforms

The application of core-shell MOFs in the development of biosensing platforms is increasing due to their unique functional and structural properties [60]. However, due to the use of the organic ligands and metal ion clusters in the fabrication of these structures, constructing a cohesive and controllable structure remains challenging. In order to overcome some drawbacks such as fine adjustment of the shell thickness, non-uniform growth of the shell, uniform distribution of NPs, reducing the toxicity of reagents, and commercialization by reducing synthesis steps, various methods have been developed to synthesize MOFs. Since different parameters such as temperature, reaction time, pressure, pH, and solvent all have a significant effect on chemical reactions for the synthesis of core-shell MOFs, reproducibility of synthesis parameters is a need for standardization [61, 62]. The most important and common production approaches to synthesize core-shell MOF nanostructure are one-pot synthesis, in situ synthesis, self-assembly, and templating. For further information regarding the synthesis of core-shell MOF nanostructures, the readers shall refer to [34, 46].

### Strategies to improve core-shell MOF performance

The very high surface area and porosity of MOFs combined with the multifunctional catalytic activities of metal compounds, have made these materials very susceptible to use in diagnostic platforms. Therefore, to increase the achievement of efficient electron transfer in core-shell MOF-based EC sensors, it is necessary to pay more attention to the selectivity, sensitivity, and reproducibility of MOFs to modulate the LOD and signal amplification.

#### Enhancement of selectivity

Although surface modification of MOFs is a potential approach in the development of biosensors with high sensitivity and selectivity, structural optimization during the manufacturing process can contribute to improved biosensing capabilities. MOFs with tunable porous structures can be used for the potential detection of biomolecules by developing pore sizes larger than that of the biomolecules. Therefore, by designing MOFs that (1) have pores larger than the size of the biomolecules, such as heterogeneous shell-core structures, or (2) have structural defects in the shell, such as porosities due to oxygen modulation, we can significantly improve the detection range of analysts. In this regard, Yang and coworkers [63] using Cu_x_O NPs@ZIF-8 containing pores with controlled dimensions on the shell, were able to detect H_2_O_2_ molecules with high efficiency and selectivity. This high selectivity was achieved even in the presence of amino acids and biological compounds, whereas metal NPs had low selectivity. Also, Luo and coworkers [64] by creating structural defects in the shell of CeO_2_-x/C nanorod with vacancies caused by oxygen modulation and increasing Ce^3+^ ion as a catalytic active site, developed a platform for indirect determination of uric acid at very low working potentials in the presence of high concentration of glucose.

#### Enhancement of sensitivity

It is well-known that decreasing the size of particles from microparticles to nanomaterials increases the sensitivity of EC sensors/biosensors. In this regard, it has been reported that nano-sized MOFs, when compared to micro-sized MOFs, increase the accessibility of electroactive sites and improve electron transport ability due to increased accessible surface area and higher porosity [65]. In addition, Lopa and coworkers [66] showed that a nano-sized metal azolate framework on the glassy carbon electrode through non-enzymatic detection significantly detected glucose in the dynamic range of 2 to 50 µM and 100 to 1800 µM with a LOD of 0.6 µM compared to bulk MOFs with a LOD of 1.46 µM [67]. In this field, it was recently determined that nano-sized urate oxidase-loaded MOF/boron nanosheets on carbon-glass electrodes improved the LOD of uric acid in the concentration range of 0.1 to 200 µM to 0.025 µM compared to other common electrodes [68]. Nonetheless, it appears that the densely adsorption of MOFs on the electrodes reduces the number of active sites and the corresponding sensor’s overall sensitivity. Hence, in order to effectively increase the sensitivity of the biosensors, it is recommended to load a single layer of MOFs on the electrodes [69]. Aside from nano-dimensions, the presence of interconnected pores can improve sensor sensitivity by facilitating electron transfer in the electrodes. For this purpose, it is necessary to investigate the chemical interactions of ligands, their exchange and mixing to create interconnected mesoporous MOFs. For example, Wang and coworkers [70] designed highly interconnected porous Cu-MOFs using an evaporation-based heteroepitaxy and self-assembly process. In this method, after the deposition of copper nanowires on paper through evaporation of dichloromethane-containing nanowires, the paper was immersed in ligand solutions and heteroepitaxial growth was induced to form Cu-MOF crystals. Their results showed that the detection sensitivity of glucose (35.9 µA/cm^2^/mm) and lactate (1690 µA/cm^2^/mm) increases significantly. However, due to the challenge of weak conductivity caused by chemical ligands and inappropriate aggregation of MOFs, the use of nano-hybrids could be a potential approach to increase the sensitivity of biosensors. In addition to increasing catalytic capabilities, nanohybrids can cause enhanced electron transfer in the developed structure. In this regard, it was reported that the electrode engineered with ultra-thin 2D MOF M-TCPP (M = Cu, Co and Ni) nanofilms (1–3 nm) and 2D MOF nanosheets with a thickness of 6–10 nm along with carbon nanosheets obtained from CNT and GO can effectively detect H_2_O_2_ with improved LOD (5 nM) at the linear range of 0.01–3.75 µM and 3.75–377.75 µM compared to 2D MOF M-TCPP [71]. Recently, in order to increase the LOD of cancer cells by sensing the generated H_2_O_2_, Huang and coworkers [72] by designing ultra-thin 2D MOF nanosheets based on hybridizing copper nanozymes with Au nanozymes in a non-aggregative form with dual enzyme-like activity were able to remarkably improve the detection of colon cancer cells. They showed that the structural changes induced through the hybridization of Cu nanozymes with Au can improve the LOD of H_2_O_2_ up to 5.6 nM with a high sensitivity of 188.1 µA/cm^2^/mM [72]. It was also found that changing the morphology of 2D Zn-MOFs hybridized with Ag NPs stimulated greater electrocatalytic activity for H_2_O_2_ detection compared to the 3D state [73]. Since Ag/2D Zn-MOFs were able to provide a large surface area and well-dispersion of NPs in their structure, they provide greater electrical conductivity compared to Ag/3D Zn-MOFs. Under optimal conditions, electrodes fabricated with Ag/2D Zn-MOF improved the LOD of H_2_O_2_ to1.67 µM with a wide range of 5.0 µM to 70 mM [73].

#### Enhancement of signal strength

The strategy of signal amplification is one of the vital approaches in improving the performance of sensors due to the very low concentration of analytes in biological samples. Despite different strategies in electric signal amplification, the use of molecular biological technologies, enzymatic methods and nanohybrids have received much attention.

One of the signal amplification strategies is the use of molecular biological technologies such as rolling circle amplification, strand displacement amplification, hybridization chain reaction (HCR), and catalytic hairpin assembly. For instance, Chen and coworkers [74] designed an MOF-based EC aptasensor composed of MIL-101@AuNPs, hemin/G-quadruplex DNA enzyme (DNAzyme), and horseradish peroxidase (HRP) for the early detection of liver cancer, which improved the LOD of HepG2 cells up to 5 cells mL^− 1^ with a wide range of 10^2^ to 10^7^ cells mL^− 1^. In addition to selective diagnosis, the designed aptasensor induced significant electrical signal amplification through synergistic cooperation of hemin/G-quadruplex DNAzyme and natural HRP. The G-quadruplex DNAzyme was prepared though the HCR method via connecting the thiolated TLS11a aptamer sequence (at the 3′ end) to two other sequences with 61 nucleotide bases. By the formation of hemin/G-quadruplex as a mimicking peroxidase the response of differential pulse voltammetry (DPV) improved significantly. Meanwhile, HRP by catalyzing hydroquinone in the presence of H_2_O_2_ effectively reduced the noise-to-signal ratio and accelerated electron transfer to improve the electrical signal [74]. In another study, it was determined that the use of a duplex hairpin probe in S1-AuNPs@Cu-MOF-based EC aptasensors not only improved the LOD of miRNA-155 to 0.35 fM with a wide linear range from 1.0 fM-10 nM but also raised hopes to amplify the electrical signal significantly [75]. In this research, it was revealed that after opening the hairpin1 (H1) structure in the presence of miRNA-155 (target) and binding to the hairpin2 (H2) structure and completing the cyclic process by miRNA-155, a significant amount of H1–H2 is formed. Then, H1-H2 duplex aggregation improved the electrical signal based on the enhancement of electron transfer in the S1-AuNPs@Cu-MOF electrode [75]. Even though molecular biological technologies offer several significant advantages, including high efficiency, programmability, biocompatibility, non-toxicity, and non-immunogenicity, they still suffer from several problems such as low HCR sensitivity and time-consuming processes. Hence, a group of researchers focused on the catalytic activity of enzymes and even pseudo-enzymes due to their controllable function and high specificity. The functional mechanism of the enzymatic approach is focused on substrate degradation like a H_2_O_2_ and noise-to-signal ratio reduction, as well as faster electron transfer by enzymatic products. For example, Li and coworkers [76] by designing glucose oxidase (GOx)/HRP@ZIF-90 as a sensor containing ovarian cancer marker and creating a competitive reaction of ATP with Zn^2+^ to break the structure of MOFs to release GOx and HRP, were able to amplify the electrical signal through the enzymatic cascade reaction. The designed aptasensor increased the LOD of CA-125 up to 0.05 pg mL^− 1^ with a wide range of 0.1 pg mL^− 1^–40 ng mL^− 1^ along with high selectivity. In another study, it was reported that the combination of tyrosinase to calcined porous carbon-based ZIF-8 containing the PSA aptamer not only increased the LOD of prostate cancer to 0.01 ng mL^− 1^ with a wide range of 0.01 to 50 ng mL^− 1^ but also significantly improves the electrical signal based on the tyrosinase activity to catalyze the oxidation of electro-inactive phenol to electro-active catechol and start the redox cycle under the influence of NADH [77]. Despite the timely and sensitive detection of biomarkers by the enzymatic approach in MOF-based sensors [78, 79], due to the complicated processes of enzyme immobilization on the electrodes and corresponding reduced enzyme, development of another strategy seems necessary. Electroactive nanohybrids with different physicochemical properties can be potentially used for the development of potential biosensing platforms via accelerating the electron transfer for signal amplification. The biocompatibility along with the fast response of nanohybrids in signal amplification has made the use of this approach interesting in the field. In this regard, Fu and coworkers [80] used AuPtRu trimetallic nanohybrids in Ce-MOF containing TSP-1 aptamer to boost the EC signal. In addition to H_2_O_2_ catalysis, the AuPtRu nanocomposite can function as a signal probe in this sensor. Therefore, the designed aptasensor has an improved LOD up to 0.13 fg mL^− 1^ with a detection range of 1 fg mL^− 1^ -10 ng mL^− 1^ [80]. Furthermore, by using Au@self-polymerized dopamine (PDA)@Fe-MOF as a biocompatible EC aptasensor, Li and coworkers [81] were able to increase the LOD of CEA up to 0.33 fg mL^− 1^ with a wide range of 1 fg mL^− 1^ −1 µg mL^− 1^. The active sites in Fe-MOF and the combination of PDA-decorated AuNPs with this platform significantly accelerated the electron transfer on the electrode surface designed for signal amplification. Recently, an EC sensor resulting from the interaction of a covalent organic framework containing nitrogen-doped graphene nanocomposite (COF-NG) with a Fe-MOF decorated with AuNPs as a capture/signal probe was designed, which is capable of high electron transfer for non-small cell lung cancer detection with LOD of 7.65 fM and a linear range of 100 fM to 100 nM [82]. Guo and coworkers [82] illustrated that although COF-NG is favorable for electron transfer due to its porous structure and good conductivity, the integration of Fe-MOF with NG-COF increased the electrical signal by inducing the reaction of Fe^3+^ with K_4_[Fe( CN)_6_].

## Electrochemical (EC) sensing strategies

The biological analyte detection by EC biosensors is mostly based on potential [voltammetry: DPV, square wave voltammetry (SWV), cyclic voltammetry (CV), and linear sweep voltammetry (LSV)], current (amperometry), and conductivity (conductometry) assays. It seems that among the above strategies, voltammetry/potentiometry has received the most interest in the field. In addition to the above findings, the use of impedance (EIS: EC impedance spectroscopy) as well as the integration of EC methods with luminescence [electroluminescence (ECL), photoelectrochemistry (PEC)] also is of a great interest. These techniques have unique features such as signal measurement, mass transfer, and specific target selection (Table 1).

**Table 1:** A summary of the mechanism, advantages and sensitivity of EC aptasensors in cancer detection

Class		Mechanism	Benefits	Sensitivity
Voltammetry	DPV	Applying amplitude potential pulses on a linear ramp potential where the selected base potential value has no Faraday reaction.	High signal-to- noise ratio, high sensitivity, low cost, portable, miniaturization capacity, using a wide range of samples, the possibility of checking solid and liquid samples, and remarkable repeatability.	Zr-MOF-on-Zn-MOF [target: protein tyrosine kinase-7 (PTK7)]: LOD of 0.66 pg mL^− 1^ [60]; Cu-MOF-RGO (target: MUC1): LOD of 7.5 pg mL^− 1^ [83]; MnO@C@AuNPs (target biomarker: MUC1): LOD of 0.31 pM [84].
	SWV	A large-amplitude differential method with wave form consisting of a symmetrical square wave that is superimposed on a base staircase potential applied to the working electrode.		HCR-Pb-MOF (target: CEA): LOD of 0.333 pg mL^− 1^ [52]; BPNSs/Fc/ZIF-67/ITO (target: MCF-7 exosomes): LOD of ∼100 particles mL^− 1^ [85]; Au/Fe-MIL-88B-NH_2_ (target: PSA): LOD of 0.13 pg mL^− 1^ [86].
	CV	The working electrode potential is ramped linearly versus time and after the set potential, the working electrode’s potential is ramped in the opposite direction to return to the initial potential.		AuNPs/Cu-MOF (target: HER2): LOD of 3.0 fg mL^− 1^ [87]; NG-PEI-COF_TAPB-TFPB_ (target: NSCLC): LOD of 7.65 fM [82]; MOF/PtNPs/G-quadruplex/hemin (target: MCF-7): LOD of 6 cells mL^− 1^ [88].
Amperometry		Measuring the analyte based on the current or potential resulting from the chemical reaction of electroactive materials on the surface of the transducer.	Low cost, good sensitivity and selectivity, and high stability.	Fe_3_O_4_@TMU-21-MWCNT (target: HER2): LOD of 0.3 pg mL^− 1^ [89].
Conductometry		The specific conductivity of an analyte is measured based on the monitoring of chemical reactions.	Fast, reliable, no reference electrode, cost-effective.	TCNQ-Cu_3_(BTC)_2_ (target: PSA): LOD of 0.06 ng mL^− 1^ [90].
Impedance	EIS	Electron transfer capacity on the electrode by applying an electric field to induce the accumulation of ions around the analytes to change the surface polarization. Then, the analytes trapped on the electrodes change the electron transfer capacity.	Wide linear range, label-free, low cost, portability, real time detection, miniaturization, and readiness for lab-on-a-chip integration.	AgNC@Apt@UiO-66 (target: CEA): LOD of 8.88 pg mL^− 1^ [91]; Cr-MOF@CoPc (target: CT26): LOD of 36 cells mL^− 1^ [92]; Zr-MOFs (target: MCF-7): LOD of 31 cell mL^− 1^ [93].
Luminescence	ECL	Response to electric current or electric field through an analyte and producing an optical phenomenon.	High range for target substrate, portability, easy storage, good reusability, low cost, rapid analysis, high sensitivity, high efficiency with MOFs, low background signal, wide dynamic response ranges, and chemical stability.	DNAzyme/gold nanorods (AuNRs)-complementary DNA (cDNA) (target: CEA): LOD of 0.036 pg mL^− 1^ [94]; Hf-TCBPE/Fc-HP_3_ (target: MUC1): LOD of 0.49 fg mL^− 1^ [95]; Zn-PTC (target: microRNA-21): LOD of 29.5 aM [96].
	PEC	Converting the chemical energy caused by the analytes into electricity under light illumination.		Zn-MOF/AuNP/AgNS (target: CA153): LOD of 0.0275 U mL^− 1^ [97]; Cu/UiO-66 (target: CEA): LOD of 0.01 ng mL^− 1^ [98].

*DPV* differential pulse voltammetry, *SWV* square wave voltammetry, *CV* cyclic voltammetry, *EIS* EC impedance spectroscopy, *ECL* electroluminescence, *PEC* photoelectrochemistry, *LOD* limit of detection

## Aptamers and aptamers-modified MOFs

### Aptamers

An aptamer is a single-stranded nucleic acid molecule with the ability to recognize specific targets and is classified based on origin, generating methods, and location of marker detection (on the tissue surface, biological secretions such as saliva, urine, and milk, and blood) [99]. Although various generating methods can be observed in the literatures [100], the use of the systematic evolution of ligands by exponential enrichment (SELEX) approach including 5 steps of binding, partitioning, washing, amplification, and conditioning [101, 102] is of great interest. The use of cell-SELEX approach is highly recommended due to the binding of the generated aptamers even with unknown membrane receptors on the cell surface [103]. In this approach, the positive selection steps include incubation, washing, and amplification of binding aptamers, while the negative selection steps remove sequences that bind to normal cells. However, the production of reliable and stable aptamers along with the aptamer selection method is still under discussion. Because some challenges of this approach, such as the complexity of some cancer cell lines, changes in protein expression, and choosing the appropriate cell line have not been fully addressed. Therefore, the clinical successes of aptamers are not comparable to those of antibodies. While, aptamers are more stable than antibodies against pH, temperature, and ionic changes [104]. Despite the wide range of aptamers produced for cancer diagnosis (Table 2), aptamers have not been notably used in clinical trials for cancer diagnosis and are being mostly studied in the laboratory. For instance, in the field of assays, Landman’s group (https://clinicaltrials.gov: NCT02957370) conducted a clinical trial for bladder cancer diagnosis based on aptamers and EC assays combined with calorimetry, the report of which is not available.

**Table 2:** A sample of aptamers used in the diagnosis of different types of cancers [105–108]

Nr.	Aptamers	Target	Cancers
1	S2.1	MUC1	Breast, lung, ovarian, pancreatic cancers, etc.
2	AFP-apt	Alpha fetoprotein	hepatocellular carcinoma
3	HB5, A30	HER2/HER3	Breast, gastric, lung, colorectal, esophageal, ovarian cancers, prostate, pancreatic, etc.
4	ESTA	E-selectin	Breast, some of metastasis
5	E0727, TuTu2231, KD1130, CL428	EGFR	Squamous cell carcinoma, breast, glioblastoma multiforme, lung, etc.
6	NOX-A12, NOX-E-36	CXCL12	Multiple myeloma, leukemia, glioblastoma multiforme
7	SYL3, EpDT3-DY647	EpCAM	Bladder, breast, colon, lung, ovarian, pancreas, prostate, etc.
8	NX-191, NX-213, Vap7, V7t1	VEGF	Lung, breast, brain, colon, pancreatic, melanoma, myeloid, gastric, etc.
9	PSMA-4-1BB	CD137	Prostate
10	xPSM-A10, A9g	PSMA	Prostate, bladder, kidney, etc.
11	ARGO100	NF-kB	Prostate, cervical, lung, breast, etc.
12	AS1411, FCL-II	Nucleolin	Leukemia, lung, renal, breast, pancreatic, etc.
13	PNDA-3	Periostin	Breast
14	ARC126, AX102	PDGF-B	Vessel, endothelial cells, retinal
15	CD40apt	CD40	Bone marrow

### Functionalization of MOF with aptamers

Functionalized MOF NPs as structural analogue of MOF, could serve as excellent signal carriers for the development of aptasensors. Indeed, abundant functional moieties on the surface of MOF pores can result in selective absorption of a large number of metal ions [109–111]. Furthermore, modified terminated DNA can be easily adsorbed on functionalized MOF-NH_2_ via the coupling and click reaction [112, 113]. For example, the first MOF developed NP-DNA biconjugate was mediated with a click reaction between dibenzylcyclooctyne-modified DNA and azide-modified MOF-N_3_ [114]. The fabricated bioconjugates with 3D spherical architecture displayed low cytotoxi**c**ity and improved stability. As a result, the stability of the signal transporters can be achieved through development of DNA-metal ions-MOF bioconjugates.

Also, Guo et al. aimed to fabricate Apt-templated AgNPs bioconjugated with Zr-MOF aptasensors through establishment of Zr−O−P interaction between MOF and the DNA strands for bifunctional EC and SPR-based detection of CEA biomarker [91]. In addition to improved biocompatibility and potential EC properties, the fabricated nanoplatform demonstrated favorable bio-affinity and excellent reproducibility. It was also shown that the developed nanoconjugates had a LOD of 8.88 and 4.93 pg·mL^–1^ derived EIS and DPV, respectively, over a wide linear range of the CEA concentration (0.01–10 ng·mL^–1^).

In general it can be suggested that functionalization of MOF with aptamers can be done through formation of Au-S bond [115], streptavidin-biotin binding [116], coordination between PO_4_^3−^ moiety and MOF [117], π-π interaction and hydrogen bonding [118], covalent binding mediated with click chemistry [119], and covalent interaction through EDC/NHS linking [120]. For further information the readers are referred to a comprehensive review reported by Liu, and coworkers [121], which described functionalization of MOF with DNA and amino acids for different applications.

### Core-shell MOF-based aptasensors

By conjugating EC aptasensors with different metallic signal tags, it has been possible to detect different biological or chemical reactions with high sensitivity [122, 123]. The development of metals-tagged aptamers is classified into two main categories: the application of pure metal NPs [124, 125], and the development of a core-shell platform with integration of metal ions into MOF NPs with a highly specific surface area [126]. Relative to the former strategy, the latter one could be utilized to develop more different types of metals-tagged aptamers owing to the modification of MOFs with multiple electroactive metal ions, including Pb^2+^, Cd^2+^, Cu^2+^, and Zn^2+^ [49].

Indeed, in order to develop aptasensors, it’s crucial to utilize appropriate substrates to tag aptamers and load sufficient metals for signal intensification. Because of its excellent adsorption properties and diverse amine and carboxyl grafting moieties, the porous MOF may be an ideal candidate for promoting the co-adsorption of substances [127, 128]. Particularly suitable candidates for the development of aptasensors are MOF NPs with good colloidal stability and a large reactive surface area. Several MOF -based aptasensors have also been developed for optical cancer biomarker detection [129–131]. Nevertheless, the application of MOF NPs to develop potential core-shell sensitive EC aptasensors for the detection of cancer biomarkers is still considered as a new area of research.

Furthermore, metal ion leakage is a significant disadvantage of metal ion adsorption on MOF as signal tags. Indeed, the interaction between metal ions and MOFs is almost entirely mediated by weak non-covalent forces, resulting in significant metal ion leaching and the inevitable generation of background (false positive) signals, reducing the specificity of this approach. Although several sensitive EC aptasensors using metallic nanotags with MOFs as carriers were developed for the detection of cancer biomarkers in recent years, it is difficult to remove the metal ion leakage mediated by MOF NPs-supported metallic signal tags. Metal ion incorporation into the MOF could be one possible approach, which is expected to result in a stable core-shell structure and low metal leakage. Indeed, when compared to traditional catalysts, MOF-based derivatives reduce metal leakage due to the protection of carbon atoms in the core-shell structure [132, 133]. Furthermore, the deposition of MOF-based nanostructures with polymers and various porous materials could be an effective method for addressing metal leakage [134].

## Application of core-shell MOFs in the detection of tumor antigens

### Prostate-specific antigen (PSA)

The tactics by utilizing synergetic core-shell MOF nanostructures as signal probes furnish a potential platform for expanding uncomplicated, rapid, and ultrasensitive dual-channel uniform aptasensors, which display an excellent prospective as promising platforms in cancer diagnosis.

Bhardwa et al. developed tetracyanoquinodimethane (TCNQ)- doped Cu-MOF, Cu_3_(BTC)_2_, adsorbed on Au electrodes, an immune-EC biosensing system, for exceptionally sensitive sensing of a PSA with a LOD of 0.06 ng mL^− 1^ and wide linearity of antigen between 0.01 and 150 ng mL^− 1^ [90]. Based on the published reports for PSA detection by other platforms, including SiO_2_ NPs (LOD = 0.76 ng mL^− 1^ ) [135], microwell SWCNT (LOD = 0.001 ng mL^− 1^) [136], graphene-modified GC (LOD = 0.008 ng mL^− 1^) [137], graphene/methylene blue nanocomposite (LOD = 0.013 ng mL^− 1^) [138], MoS_2_ (LOD = 0.001 ng mL^− 1^) [139], graphene/Au (LOD = 0.59 ng mL^− 1^) [140], GOQDs (LOD = 0.0003 ng mL^− 1^) [141], MXene-Au-MB (LOD = 0.00008 ng mL^− 1^) [142], DNA tetrahedron structural probes (TSPs)-Au nanoflowers (NFs)-modified screen-printed electrodes (SPEs) (LOD = 0.2 ng mL^− 1^) [143], the LOD of this MOF-based biosensor is comparable with others. However, other platforms, such as carbon QDs-AuNPs with a LOD of 2 fg mL^− 1^ [144] and hierarchical SiO_2_@MoS_2_ nanostructures with a LOD of 2.5 fg mL^− 1^ [25], have recently demonstrated significantly lower LOD than that of TCNQ- doped Cu-MOF. Therefore, optimization of fabricated core-shell platforms may be a potential strategy for biomarker detection [145, 146].

Table 3 also summarizes the application of core-shell MOF NPs in the design of apta/biosensor for ultrasensitive PSA detection.

**Table 3:** Core-shell MOF-based EC apta/sensor for ultrasensitive PSA detection

Platform	LOD	Linear range	Detection type	Refs.
AgNC@Apt@UiO-66	8.88 and 4.93 pg·mL^–1^	0.01–10 ng·mL^–1^	EIS and DPV	[91]
Au-hemin-Mil-DNAzyme	0.058 ng mL^− 1^	0.5 to 500 ng mL^− 1^	EIS	[147]
PdNPs@Co-MOF	0.03 pg mL^− 1^	0.01 fg.mL^–1^ to 50 ng. mL^–1^	EIS	[148]
Pd@hollow Zn/Co core–shell ZIF67/ZIF8	0.78 pg mL^− 1^	5 pg mL^− 1^ to 50 ng mL^− 1^	EIS	[149]

### Carbohydrate antigen 15 − 3 (CA15-3)

**Fig. 1:**
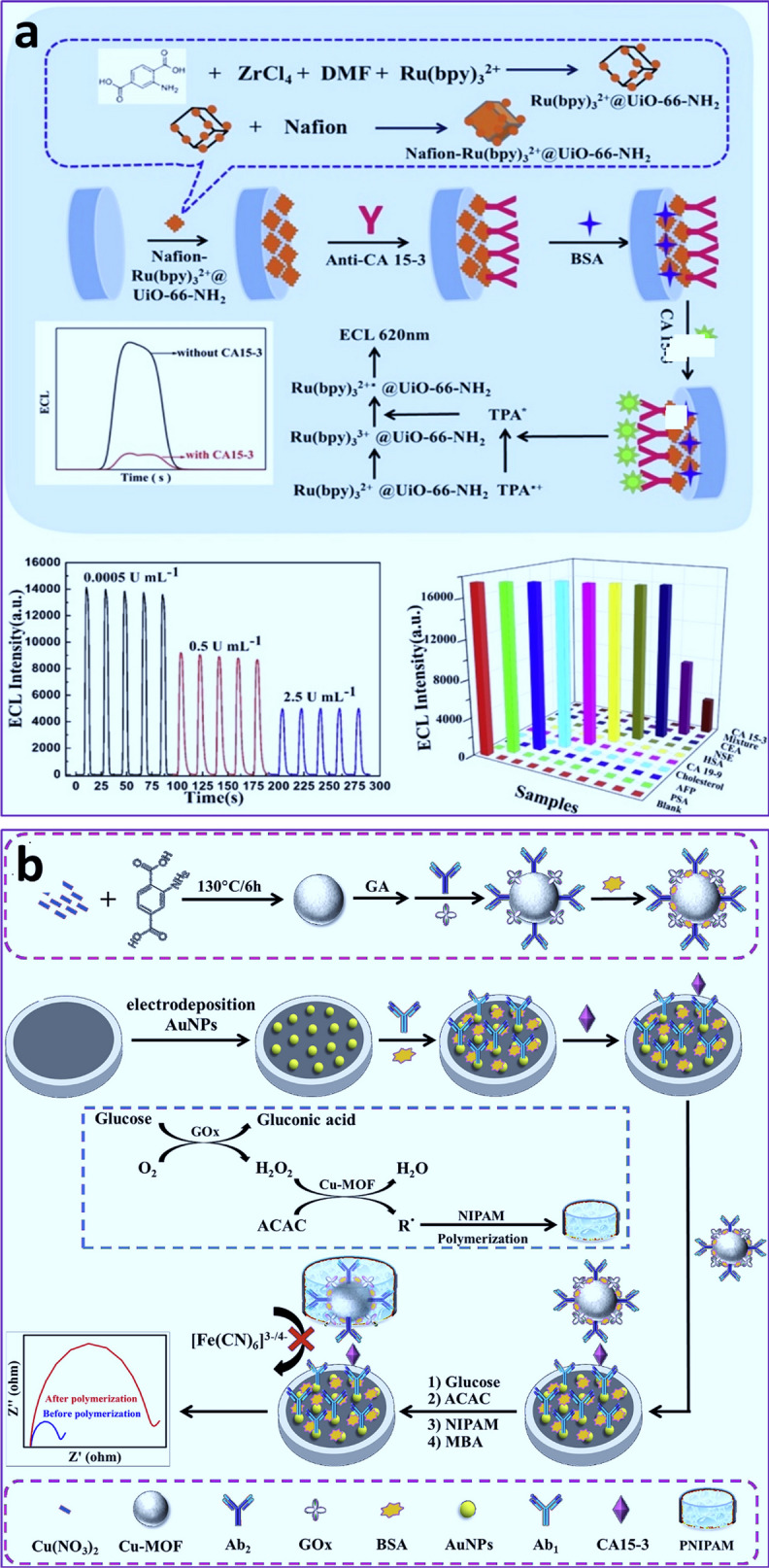
**a** Schematic illustration of an ELC immunoassay for CA 15−3 detection by using Ru(bpy)_6_^2+^-functionalized amino-coated UiO-66 MOF NPs [150]. Reprinted with permission from Ref. [150], copyright 2019, Elsevier. **b** Schematic illustration of immune-based MOF EIS biosensors for cascade catalysis-initiated radical polymerization-stimulated signal intensification for CA15-3 detection [151]. Reprinted with permission from Ref. [151], copyright 2019, Elsevier.  *GOx* Glucose oxidase, ***ACAC*** acetylacetone, *PNIPAM* poly (N-isopropylacrylamide)

CA15-3 could represent a potential tumor marker in several types of cancers such as ovarian, lung, and prostate, as well as benign breast cancer. Xiong and and coworkers [150] developed an ECL immunoassay for CA 15−3 detection by using Ru(bpy)_6_^2+^-functionalized amino-coated UiO-66 MOF NPs. Ru derivatives were used as a luminescent probe, with UiO-66-NH2 acting as a carrier and Nafion as a fixer (Fig. 1a). Also, the covalent conjugation of CA 15 − 3 Ab was mediated by amide reaction. It was discovered that the ECL signal was apparently quenched after the CA15-3 marker interacted with the immunosensor, and the LOD was in the range of 5 × 10^− 4^ to 5 × 10^2^ U mL^-1^ and a LOD of 1.77 × 10^− 5^ U mL^-1^, which was applicable in real samples [150]. Also, a signal amplification procedure mediated by initiated radical polymerization activated with coupling cascade catalysis was introduced for ultrasensitive sensing of CA15-3 through EIS immune-based assay [151]. As immune-based probes, Cu-MOF as a peroxidase-mimic enzyme in combination with Ab and GOx, generation of H_2_O_2_, was able to initiate radical polymerization via cascade catalysis. Indeed, resistance values improved following the interaction of H_2_O_2_ with acetylacetone catalyzed by Cu-MOF and the formation of acetylacetone radicals-based poly N-isopropylacrylamide (Fig. 1b). This biosensor detected CA15-3 at detection ranges ranging from 10 µU mL^− 1^ to 100 mU mL^− 1^ with a low LOD of 5.06 µU mL^− 1^ for CA15-3 [151]. It should be noted, however, that both platforms serve as immunobiosensors that rely on Abs, which present some challenges in biosensor development, such as partial denaturation and orientation. As a result, the drawbacks of Ab-based biosensors can be addressed by using another strategy, aptasensors, which needs further investigations in the future studies.

### Carcinoembryonic antigen (CEA)

**Fig. 2:**
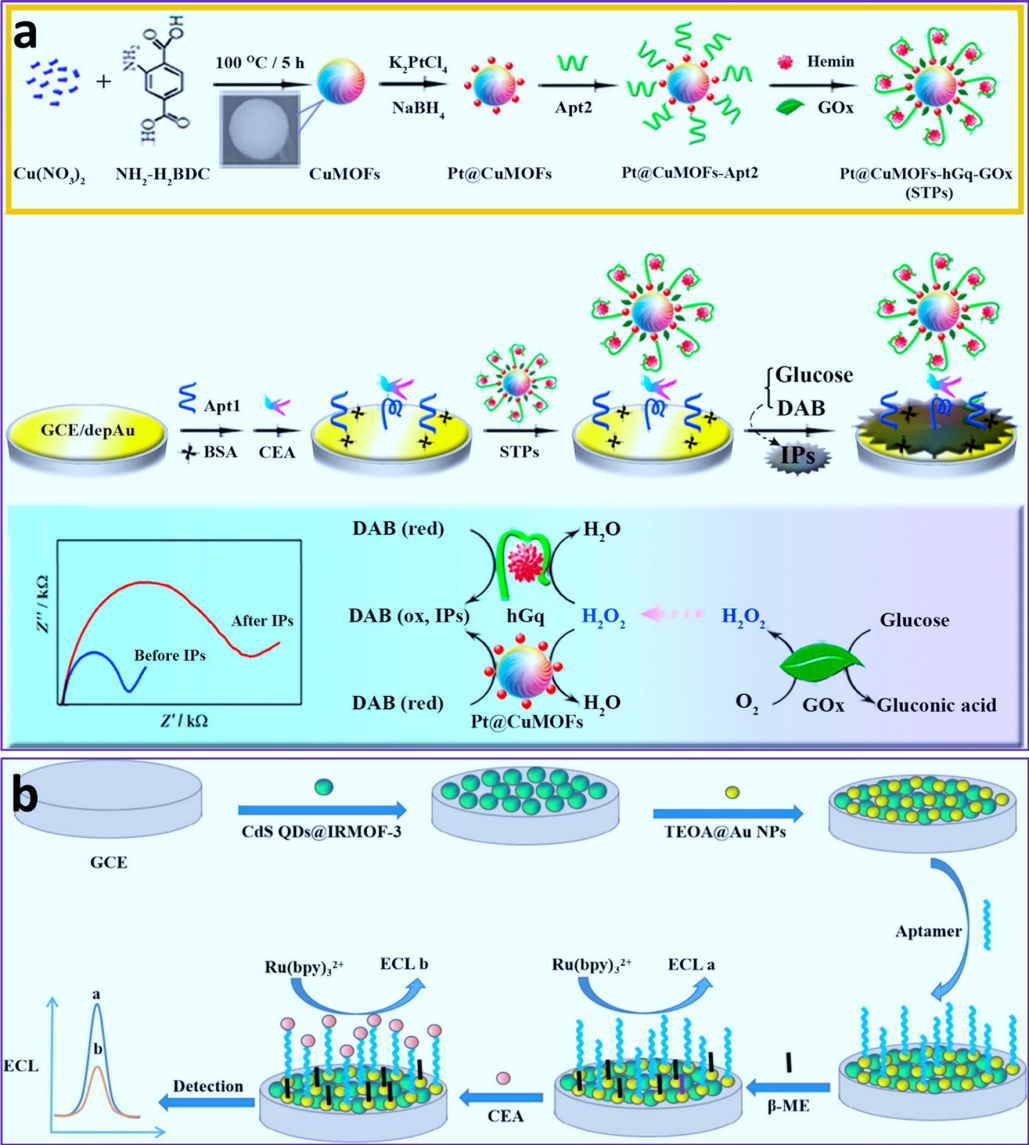
**a **Schematic illustration of core-shell MOF-based EIS aptasensor for cascade catalysis-initiated radical polymerization stimulated signal intensification for CEA detection [152]. Reprinted with permission from Ref [152], copyright 2017, Elsevier. **b** Schematic illustration for the fabrication of CdS QDs@MOF and TEOA@Au aptasensor for CEA detection [154]. Reprinted with permission from Ref. [154], copyright 2022, Elsevier. *IPs* Insoluble precipitates, *DAB* 3,3-diaminobenzidine, *TEOA* triethanolamine

A high level of CEA could indicate several types of cancer, including colorectal, prostate, ovarian, lung, thyroid, and liver cancer. Zhou and coworkers [152] described a GOx-mediated cascade catalysis for the development of an ultrasensitive EIS aptasensor catalyzed by Pt@MOF NPs and hemin/G-quadruplex (hGq) as peroxidase-mimic enzyme for the oxidation of conductive 3,3-diaminobenzidine (DAB) and the generation of insoluble precipitates with minimum conductive properties, with a low LOD of 0.023 pg mL^− 1^ toward CEA (Fig. 2a) [152]. Guo and coworkers [91] also reported an AgNP@Apt@Zr-MOF platform for the development of bifunctional EC and optical aptasensors toward CEA with LODs of 4.93–8.88 pg·mL^–1^ and 0.3 pg·mL^–1^, respectively and a broad linear range of the CEA concentration (0.01–250 ng·mL^–1^) [91]. Therefore, the synthesis route of MOF, structure, and type of MOF can play a key role in the sensitivity of MOF-based biosensors in detection of biomarkers. Also, by using MOFs as a nanocarrier of EIS active materials (methylene blue) along with controllably assembled DNA, gatekeeper, a smart platform based on target (CEA)-guided cascade boosted release of methylene blue was developed which was able to detect CEA with a LOD of 16 fg mL^− 1^ and broad linear range of 50 fg mL^− 1^ to 10 ng. mL^-1^ [153]. Furthermore, Li and coworkers [81] developed an EC aptasensor composed of self-polymerized dopamine modified Au coordinated with Fe-MOF (abbreviated as: Au@PDA@Fe-MOF) for the detection of CEA with improved sensitivity, several active sites, good biocompatibility, and potential selectivity derived from several − COOH groups and Fe^3+^ sites in porous and on the surface of Fe-MOF, respectively. Then, NH_2_-modified CEA-selective aptamer and redox PDA along with Fe-MOF could accelerate the electron transfer for dual signal intensifying [81]. The developed aptasensor had a broad antigen detection range from 1 fg mL^-1^ to 1 µg mL^-1^ with a LOD of 0.33 fg mL^-1^. Moreover, a label-free ECL aptasensor for ultrasensitive detection of CEA was developed based on CdS QDs-modified MOF and triethanolamine-modified AuNPs as bi-coreactants of Ru(bpy)_3_^2+^ ECL platform, as well as a carrier for aptamer (Au–S bond) (Fig. 2b) [154].

### Human epidermal growth factor receptor-2 (HER2)

**Fig. 3:**
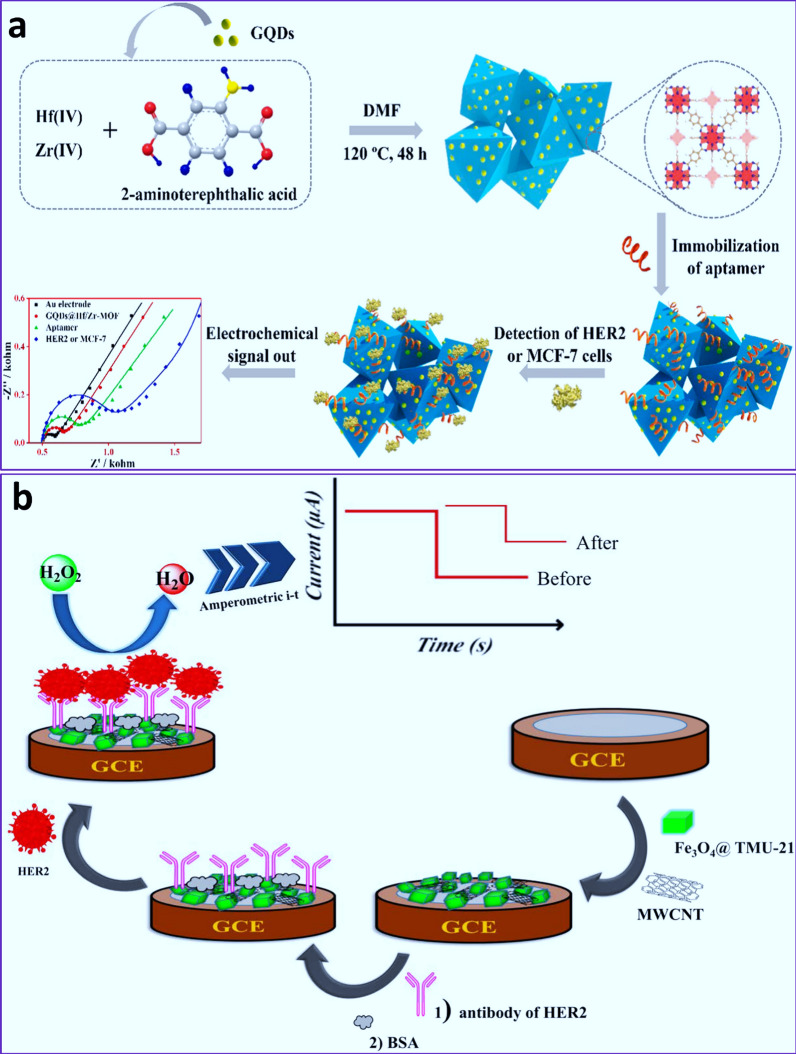
**a** Schematic illustration of the synthesis of CDs@ZrHf-MOF-based aptasensor for the detection of HER2 [155]. Reprinted with permission from Ref. [155], copyright 2019, Elsevier. **b** Schematic illustration of the synthesis of Fe_3_O_4_@TMU-21-MWCNT-based immunosensor for the detection of HER2 [89]. Reprinted with permission from Ref. [89], copyright 2020, Elsevier. *CDs* Carbon dots, *BSA* bovine serum albumin

Approximately 20–30% of breast tumors upregulate the expression of HER2. The bimetallic ZrHf-MOF embedded with carbon dots (abbreviated as CDs@ZrHf-MOF) was used as a potential systems for the detection of HER2 in MCF-7 cancer cells through EC aptasensor with a LOD of 19 fg mL^−1^ (Fig. 3a) [155]. It was seen that LOD of other biosensors for HER2 detection including Au nanostructured screen-printed graphite [156], CuO NPs [157], AuNP-based rolling circle amplification [158], ferrocene-labeled DNA/Au [159], polycytosine DNA [160], aptamer-based interdigitated electrode [161], antiHER2/APTMS-Fe_3_O_4_ [162] were 6.0 pg mL^− 1^, 0.956 pg mL^− 1^, 80 fg mL^− 1^, 4.9 ng mL^− 1^, 0.5 pg mL^− 1^, 0.1 ng mL^− 1^, 0.02 pg mL^− 1^, respectively.

Also, a magnetic Fe_3_O_4_@ TMU-21-MWCNT with redox activity toward H_2_O_2_ was used as a potential immunosensor against HER2 [89]. Indeed, with HER2 biomarker the amperometric current of H_2_O_2_ changes, which could be a sign of antigen–Ab interaction on the electrode with a linear range of 1.0 pg mL^− 1^-100 ng mL^− 1^ and LOD of 0.3 pg mL^− 1^ (Fig. 3b) [89]. Although the detection of HER2 is well-correlated with human serum samples, the use of immune-based biosensors due to some challenges such as low stability and sensitivity may limit their clinical application. For example, it has been reported that aptasensors outperform immunosensors in terms of sensitivity, reusability, and storability against HER2 detection [163].

It was shown that the LOD of Fe_3_O_4_@TMU-21-MWCNT for the detection of HER2 via amperometric method was significantly lower than those of Hyd–AuNP − Apt (SWSV, 37 pg mL^− 1^) [124], Au NPs-modified disposable screen-printed carbon electrodes (impedimetric, 0.01 ng mL^− 1^) [164], inkjet printed Au working 8-electrode array (amperometry, 12 pg mL^− 1^) [165], streptavidin-alkaline phosphatase (LSV, 0.16 ng ml^− 1^) [166], Ab2-PbS QDs (SWV, 0.28 ng mL^− 1^) [167], and CdSe@ZnS (DPV, 2.10 ng ml^− 1^) [168]. However, some other biosensor platforms have been reported to outperform Fe_3_O_4_@ TMU-21-MWCNT sensor for the detection of HER2 such as anti-HER2 conjucated mesoporous ZnO nanofibers (EIS, 185 fg ml^− 1^) [169], hierarchical composite of porous graphene and TiO_2_ nanofibers (EIS, DPV, 185 fg ml^− 1^) [170], and Fe_3_O_4_–Au NPs–AgNPs (DPV, 20 fg ml^− 1^) [162].

### Cancer antigen 125 (CA-125)

**Fig. 4:**
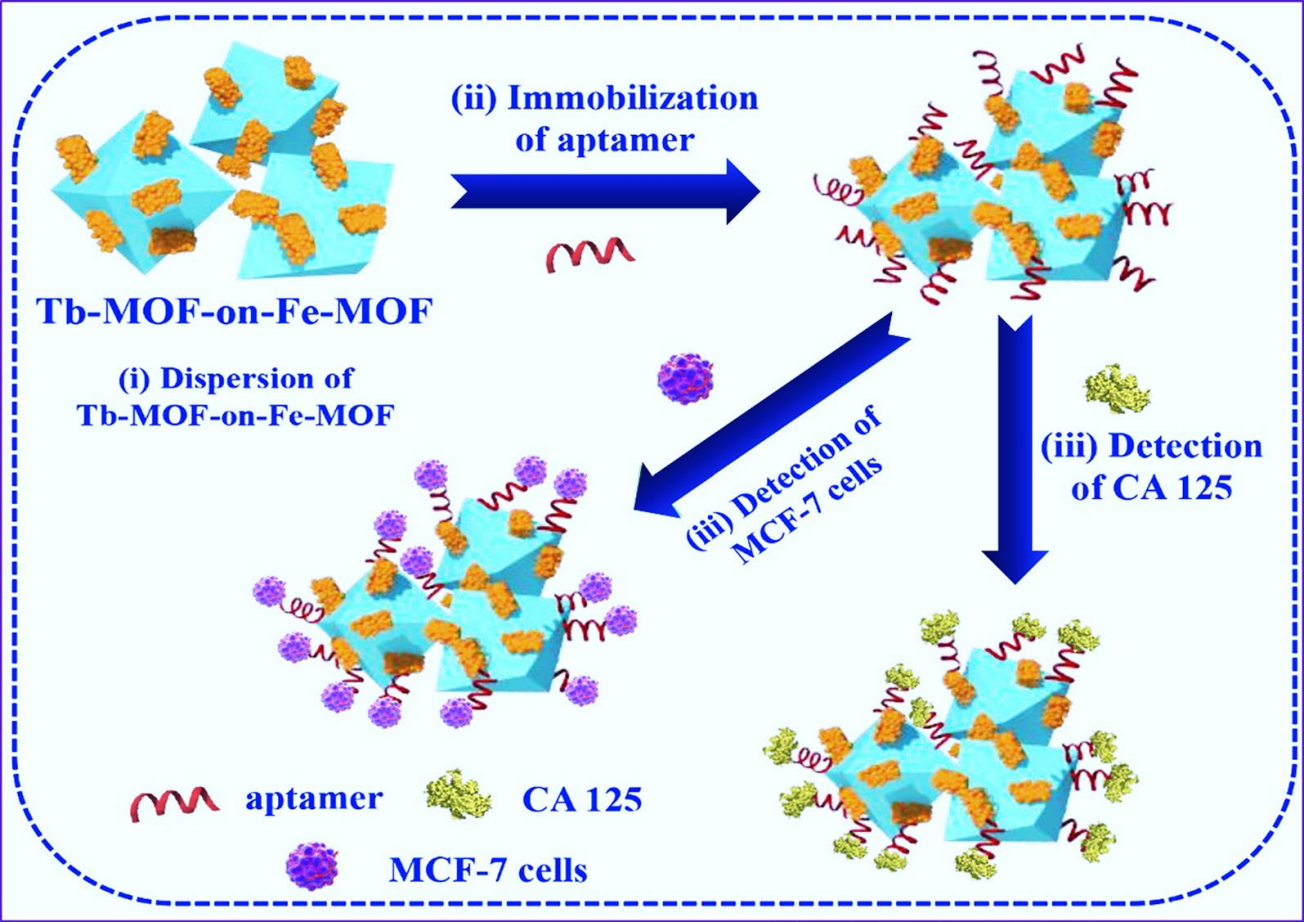
Schematic illustration of core-shell bimetallic TbFe-MOF for the immobilization of the aptamer strands for concurrently sensing CA-125 and living cancer cells [177]. Reprinted with permission from Ref. [177], copyright 2019, Elsevier

The CA-125 detection can be applied to detect early signs of ovarian cancer. Serval biosensors such as FA-HCl-doped polyaniline-chitosan-Ag-Co3O4 nanosheets [171], MoS_2_-Au-nanoflowers [172], Au NP-ZnO nanorods [173], graphene polyaniline [174], 3D Au electrode [175], benzothiophene derivative [176] have been developed for sensitive EC determination of CA-125 with an LOD ranging from 0.25 pg mL^− 1^ to 2.5 ng mL^− 1^. Regarding the apparent biosensing properties, advanced EC performance, and exceptional biocompatibility of Fe-/Tb-MOF, as well as fluorescence properties of Tb-MOF, the heteroarchitectured core-shell bimetallic TbFe-MOF could be developed and recruited as an advance system to immobilize aptamer strands for concurrently sensing cancer biomarkers and living cancer cells (Fig. 4a) with a LOD of 0.000058 U·mL^− 1^ via EIS detection method [177]. The developed biosensor showed a potential binding of aptamer with CA-125 biomarker evidenced by an apparent decrease in current (preventing electron transfer at electrode-solution interface) and an increase in ΔEp [177].

It was found that other biosensors including microfluidic origami device [178], mercaptopropionic acid/AuNP@SiO_2_/CdSe QD [179], multi-functionalized g-C_3_N_4_ [180], Au nanostructures [181], phosphoserine imprinted CNT nanosensor [182], chitosan-AuNPs/multiwall carbon nanotube/GO [183] with different detection methods show a LOD in the range of 0.0016 to 5.5 U·mL^− 1^, which is not comparable with that of TbFe-MOF [177].

In another study, tricopper benzene-1,3,5-tricarboxylate (CuBTC) CuBTC@MoS_2_-AuNPs/CA125 Ab-functionalized electrodes were developed, where MoS_2_ and AuNPs were used for enhancing the electron transfer capability and attachment of Ab, respectively. Therefore, based on the significant synergistic effect-derived EC signal, the modified CuBTC@MoS_2_-AuNPs/CA125 Ab electrode was able to detect CA125 with a LOD of 0.0005 U mL^− 1^ and a broad linear range of 0.5 mU mL^− 1^ to 500 U mL^− 1^ by DPV [184]. It was shown that other EC immunosensors including mercaptopropionic acid/AuNP@SiO_2_/QD [179] and chitosan-AuNP/MWCNT/ GO [183] show LODs (U mL^− 1^) of 0.0016 and 0.002, respectively for CA-125, which were significantly higher than that of CuBTC@MoS_2_-AuNPs/CA125 Ab [184]. However, it has been reported that Fe_3_O_4_@g-C_3_N_4_-based EC immunosensor with a LOD of 0.0004 U mL^− 1^ [180] shows comparable outcomes with CuBTC@MoS_2_-AuNPs/CA125 Ab [184].

### Cytokeratin 19 fragment (CYFRA21-1)

**Fig. 5:**
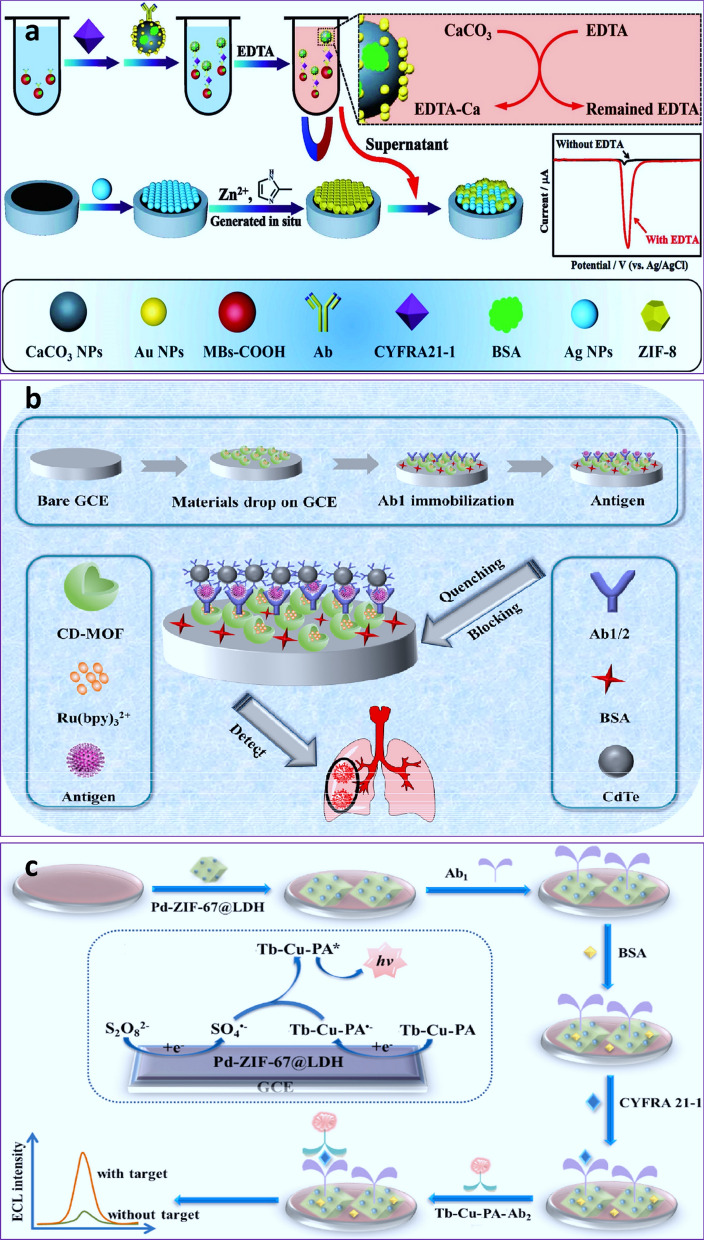
**a** Schematic illustration of the complexation competition approach for the detection of CYFRA21-1 based on ZIF-8 [189]. Reprinted with permission from [189], copyright 2020, Elsevier. **b** Schematic illustration of the synthesis of the CD-MOF@Ru(bpy)_3_^2+^ nanostructure for detecting CYFRA21-1 using an ECL strategy [190]. Reprinted with permission from Ref. [190], copyright 2021, Elsevier. **c** Schematic illustration of immunosensor and ECL detection of CYFRA21-1 [191]. Reprinted with permission from Ref. [191], copyright 2022, Elsevier. *CD* Cyclodextrin, *Pd-ZIF-67@LDH* Pd NPs functionalized Ni-co layered double hydroxide, *MBs* magnetic beads

The CYFRA21-1 is known as an important tumor marker, particularly for lung cancer. Different EC-based platforms such as BSA/Anti-Cyfra-21-1/ncCeO_2_-RGO/ITO [185], GCE/Fe_2_N/rGO/Au-HWR/Ab1/BSA/CYFRA21-1/Ab_2_-apoFt@Ir (ppy)_3_ [186], Au-pThi/anti-CYFRA21-1/CYFRA21-1/anti-CYFRA21-1/Au/3D-G/GCE [187], and GCE/nafion-AuNPs/Ab_1_/BSA/CYFRA21-1/Ab_2_-TB-AuNPs@MoS_2_@Ti_3_C_2_T_*x*_ [188] with LODs (pg mL^-1^) of 0.625, 0.43, 180, and 0.03 have been reported for the detection of CYFRA21-1 marker. Literature survey showed that, although there is no report on the simultaneous use of aptamers and MOF for EC-based detection of CYFRA21-1, there were several reports on the EC immunoassay. For example, Xu and coworkers [189] aimed to develop a feasible and ultrasensitive immunosensor based on complexation competition reaction between CaCO_3_ NP-Au modified with Ab, ZIF-8, and EDTA for EC detection of CYFRA21-1. Excessive EDTA was used in the complexation reaction with CaCO_3_ NPs and for etching ZIF-8 and AgNPs (signal amplifier) and ZIF-8 (signal silencer) onto the electrode surface. Because of the EDTA-based destruction of ZIF-8, the LSV signal of AgNPs was amplified (Fig. 5a) [189]. As a result, the amount of CYFRA21-1 modified with magnetic beads was quantified by analyzing the current signal of AgNPs, with a LOD of 3.175 fg mL^− 1^ and a broad detection range of 10 fg mL^− 1^ to 1 µg mL^− 1^. Also, a Ru(bpy)_3_^2+^ encapsulated cyclodextrin-based MOF with good biocompatibility was developed for ultrasensitive ECL detection of CYFRA21-1 in serum and A549 lung cancer cells, with a LOD of 0.006 ng mL^− 1^ and a broad liner range of 0.1–50, 50–200 (ng mL^− 1^) (Fig. 5b) [190]. Furthermore, the sandwich-typed Tb-Cu-m-phthalic acid (PA) lanthanide MOF immunoplatform was used as a potential ECL-based platform for the detection of CYFRA21-1, where captured Ab was immobilized on Pd NPs functionalized Ni-Co layered double hydroxide (Pd-ZIF-67@LDH) nanostructures with high electrocatalytic activity for intensifying the ECL signal (Fig. 5c) [191]. The developed platform showed a broad linear range of 0.01–100 ng mL^− 1^ and a low LOD of 2.6 pg mL^− 1^ [191]. Comparison between ECL immunosensor and other strategies for CYFRA 21 − 1 detection indicated that ECL detection method provide a lower LOD (0.0026 ng mL^−1^) in comparison with other EC detection methods with LOD in the range of 0.043–0.122 ng mL^−1^ [192–194].

### Other tumor markers

Zhou and coworkers [60] reported the synthesis of two types of bimetallic ZnZr-based MOFs through MOF-on-MOF strategy and used them as a platform for immobilization of aptamer to develop a sensitive aptasensor to detect the cell membrane PTK7 as a tumor marker. It was seen that the developed core-shell hybrid bimetallic MOF was able to detect PTK7 marker with a LOD of 0.84 pg mL^− 1^ and 0.66 pg mL^− 1^ with detection range of 0.001 − 1 ng mL^− 1^ through EIS and DPV, respectively.

Therefore, it was assumed the Zn-MOF-on-Zr-MOF could serve as a potential platform to provide higher signal output relative to the Zr-MOF-on-Zn-MOF platform [60]. Then, it was seen that Zn-MOF-on-Zr-MOF architecture decorated with aptamer can show higher sensitivity for the detection of PTK7 relative to structure-switching aptamer [195], [Ir(pbi)2(5,5-dmbpy)]PF6 [196], DNA-AuNPs/aptamer/AuNPs/Nf [197], DNA-AgNCs [198] with fluorescence, luminescence, DPV, and fluorescence detection methods, respectively. Indeed, it was discovered that the LOD of the above-mentioned method was in the range of 0.048-13 ng mL^− 1^, whereas the LOD of Zn-MOF-on-Zr- MOF was in the range of 0.66–0.84 pg mL^− 1^. Also, early detection of platelet-derived growth factor-BB (PDGF-BB), an important protein marker upregulated in tumor cells, can provide useful information for treatment of a broad range of cancers. Based on this theory, Li and coworkers [199] developed a potential core–shell nanostructure containing Cu-based MOF (Cu-MOFs) as well as COFs (TpBD) to be used as a potential platform for fabrication of an aptasensor for the detection of PDGF-BB. The Cu-MOFs (core) and TpBD (shell) were used in signal amplification and immobilization of PDGF-BB biomarker with a LOD of 0.034 pg mL^− 1^ within the detection ranges of 0.0001 to 60 ng mL^− 1^ [199]. Therefore, it was claimed that concurrent application of MOFs and COFs, as well as the adsorption of tumor marker-specific aptamer via different hydrophobic and hydrophilic interactions, could result in the fabrication of a potential core-shell-based aptasensor that can be used as a productive strategy to fabricate a biosensor for the feasible, accurate, and selective determination of a specific biomarker in clinical settings.

## Conclusions and future perspectives

Promising results of EC aptasensors based on core-shell MOFs have enabled us to detect cancer biomarkers in a more accurate and sensitive manner in comparison with standard approaches. This category of aptasensors with the high loading of probes due to the increase of the surface-to-volume ratio and interconnected cavities along with the resistance of probes against destruction, were able to improve the selectivity, sensitivity, LOD, mass transfer and signals amplification of MOFs-based EC aptasensors compared to other electrodes. There are, however, some challenges needed to be addressed to make core-shell MOD-based EC aptasensors effective in clinical settings, including:

A positive or false negative signals: Because MOFs have a porous structure with high accessible surface areas, non-specific adsorption of biomaterials can negatively influence the assay. Thus, being able to manipulate the pore structure of MOFs during growth is a crucial requirement. Increasing the binding sites of target analytes by modifying the functional groups on surfaces seems like an effective way to reduce the binding of other biomaterials.

Biocompatibility: The catalytic activity outcomes reveal a significant increase in the catalytic performances of monometallic MOFs after incorporation of another metal or carbon atom in the same platform as well as an excellent enhancement in EC properties. However, commercializing this type of aptasensor is challenging because of biocompatibility and biodegradability issues.

Controllable dimensions and shapes: As expressed in many reports, the effects of morphology and size of MOFs on their functions have not been studied analytically, which can limit their practical application. Since nanoscale dimensions with polyhedral morphology can affect the performance of sensors [200, 201], it appears that producing a core-shell MOF-based EC aptasensor with uniform distribution improves sensitivity and accuracy mediated by boosting electron transfer.

Complex and expensive production: One of the most important challenges in the development of core-shell MOF-based EC aptasensors is the costly, complicated production on large scale, as well as insufficient accuracy and efficiency of these platforms in clinical applications due to the use of various ligands and aptamers in one platform compared to standard biosensors. Because detecting cancer with a single aptamer is fraught with false positive test results, the use of multifunctional core-shell MOFs-based EC aptasensors with multiple aptamers to simultaneously detect two or three targets is currently considered a potential strategy to address this issue.

Reproducibility and stability: Due to a lack of complete understanding of MOF growth mechanisms on electrode surface, designing electrodes with the controllable pores and structures that provide stability, electron transfer, and uniform electrocatalytic performances for clinical diagnostics is not possible. The integration of multiple NPs in MOFs, which can improve electron conductivity and catalytic activities, may provide some merits to overcome this concern. However, due to the lack of complete understanding of the synergistic effect of NPs with MOF and their effect on the loading of aptamers along with the excessive stability of NPs in the environment, it faces further challenges when it comes to their use in clinical settings.

Large-scale production: Potential immobilization and conjugation of aptamers on core-shell MOFs is a critical concern that should be properly addressed for the clinical diagnostics of biomarkers. The lack of comprehensive studies on the stability of core-shell MOF-based EC aptasensors, as well as their effect on diagnostic accuracy, has rendered the large-scale production of analogous sensors impossible. In fact, it is complicated to engineer the conformation, configuration, density, and stability of aptamers upon conjugation with core-shell MOF NPs. While, the interaction of aptamers on the surfaces and probably their spatial structure changes are a function of the inherent behavior of the nucleic acid sequences and their different interactions with the surface properties of the platform. It’s undeniable that these effects can greatly reduce, if not stop, the accuracy and sensitivity of diagnosis in the clinical applications. A theory-based approach combined with computational methods can provide a more accurate assessment of the aptamer 3D configuration on the core-shell MOF NPs in the initial phase.

Overall, it appears that core-shell MOF-based EC aptasensors will become increasingly practical in clinical applications as a result of the efforts made to address the challenges.

## Data Availability

No data was used for the research described in this review article. This paper presents figures that do not belong to the authors; they have been reprinted with permission from the cited references.
